# Theory and Practice of Glucocorticoids in COVID-19: Getting to the Heart of the Matter—A Critical Review and Viewpoints

**DOI:** 10.3390/ph16070924

**Published:** 2023-06-25

**Authors:** Francesco Salton, Paola Confalonieri, Gianfranco Umberto Meduri, Lucrezia Mondini, Liliana Trotta, Mariangela Barbieri, Chiara Bozzi, Chiara Torregiani, Selene Lerda, Mattia Bellan, Marco Confalonieri, Barbara Ruaro, Stefano Tavano, Riccardo Pozzan

**Affiliations:** 1 Pulmonology Unit, Department of Medical Surgical and Health Sciences, University Hospital of Cattinara, University of Trieste, 34149 Trieste, Italyriccardo.pozzan@outlook.it (R.P.); 2 Department of Medicine, Division of Pulmonary, Critical Care, and Sleep Medicine, University of Tennessee Health Science Center, Memphis, TN 38163, USA; 3 Business School, University of Milano, 20149 Milano, Italy; 4 Department of Translational Medicine, Università del Piemonte Orientale (UPO), 28100 Novara, Italy; 5 Center for Autoimmune and Allergic Disease (CAAD), Università del Piemonte Orientale (UPO), 28100 Novara, Italy; 6 A.O.U. Maggiore della Carità, 28100 Novara, Italy

**Keywords:** glucocorticoids, COVID-19, systemic inflammation, cytokines

## Abstract

Prolonged, low-dose glucocorticoids (GCs) have shown the highest efficacy among pharmacological and non-pharmacological treatments for COVID-19. Despite the World Health Organization’s recommendation against their use at the beginning of the pandemic, GCs at a dose equivalent to dexamethasone 6 mg/day for 10 days are now indicated in all COVID-19 cases who require respiratory support. However, the efficacy of the intervention depends on the timing of initiation, the dose, and other individual factors. Indeed, patients treated with similar GC protocols often experience different outcomes, which do not always correlate with the presence of comorbidities or with the severity of respiratory involvement at baseline. This prompted us to critically review the literature on the rationale, pharmacological principles, and clinical evidence that should guide GC treatment. Based on these data, the best treatment protocol probably involves an initial bolus dose to saturate the glucocorticoid receptors, followed by a continuous infusion to maintain constant plasma levels, and eventually a slow tapering to interruption. Methylprednisolone has shown the highest efficacy among different GC molecules, most likely thanks to its higher ability to penetrate the lung. Decreased tissue sensitivity to glucocorticoids is thought to be the main mechanism accounting for the lower response to the treatment in some individuals. We do not have a readily available test to identify GC resistance; therefore, to address inter-individual variability, future research should aim at investigating clinical, physiological, and laboratory markers to guide a personalized GC treatment approach.

## 1. Introduction

Glucocorticoids (GCs) are endogenous modulators of systemic inflammatory processes, besides exhibiting several other genomic and non-genomic effects. Cytokines released during the innate immune response (e.g., IL-6 and IL-1β) trigger the hypothalamic-pituitary-adrenal axis to release GCs, which primarily operate by binding the ubiquitous cytoplasmic glucocorticoid receptor (GR). GR dimerizes and enters the nucleus after ligand-receptor interaction. There it binds with transcription factors to downregulate proinflammatory pathways, including c-Jun and NF-kB [1,2].

Physiological GC response is central in acute conditions such as sepsis and acute respiratory distress syndrome (ARDS), in which dysregulated inflammation is the primary driver of multi-organ failure and mortality. Indeed, the endogenous glucocorticoid-mediated anti-inflammatory activity may be relatively insufficient during these hyper-inflammatory states, providing a rationale for exogenous GC administration. This phenomenon, known as critical illness-related corticosteroid insufficiency (CIRCI), is thought to be caused by three major pathophysiologic events: imbalance of the hypothalamic-pituitary-adrenal (HPA) axis, abnormal cortisol metabolism, and peripheral tissue resistance to glucocorticoids [3].

Since the early 1980s, strong evidence favoured the use of low GC doses in sepsis, severe bacterial pneumonia, and ARDS with a positive effect on mortality, radiological progression, clinical instability, and the need for mechanical ventilation [4,5]. The definition of low doses has been debated, but there is agreement around a dose of ≤1 mg of methylprednisone equivalent/Kg/day for 8–14 days to be regarded as low in severe acute illnesses, as opposed to chronic disorders which often require long-term treatment courses [3]. Contrarily, short courses (≤3 days) of really high doses (10–30 mg of prednisone equivalent/Kg/day) of GCs have been shown to increase mortality in both septic shock and ARDS due to inflammatory rebound after discontinuation [6]. In 2018, a joint consensus statement of the Society of Critical Care Medicine and of the European Society of Intensive Care Medicine [7] recommended treating patients with early moderate to severe ARDS with an intravenous (IV) continuous infusion of methylprednisolone 1–2 mg/kg/day for 14–28 days, titrated on clinical circumstances. Before the COVID-19 pandemic breakout, data coming from a Cochrane metanalysis provided definitive evidence that prolonged low-dose GC treatment was associated with significant mortality benefits in community-acquired pneumonia and ARDS [8].

This notwithstanding, the World Health Organization (WHO) recommended against the administration of GCs in SARS-CoV-2-related pneumonia at the beginning of the COVID-19 pandemic. This was due to observational data obtained during the SARS-CoV and MERS-CoV pandemics, which correlated GC use with a higher plasma viral load, a longer time for viral shedding, a longer length of hospital stay, a higher probability of bacterial superinfection, and a higher mortality [9,10]. However, higher doses of GCs were associated with prolonged viral shedding compared with lower-dose GCs in a dose-response fashion in these studies [11,12,13], coherently with former ones [14,15].

Indeed, the initial WHO position was changed towards a strong recommendation in favour of GCs after the massive body of evidence, which followed the RECOVERY trial, demonstrating the clear effect of GCs on mortality reduction in moderate and severe COVID-19 [14,16]. The RECOVERY (Randomized Evaluation of COVID-19 Therapy) protocol was developed in early 2020 when no treatments for COVID-19 had been approved yet. This protocol describes a randomized study among adults hospitalized with confirmed COVID-19, randomly assigned between different treatment options (initially no add-on treatment vs Lopinavir-Ritonavir vs Interferon β vs low dose corticosteroids), which could increase in number while knowledge about the virus had increased over the months [14,16]. The primary follow-up timeline was set at 28 days after admission. The main outcomes considered were in-hospital death, discharge, and the need for invasive mechanical ventilation (IMV; intubation or tracheostomy) [14,16].

This review summarizes the current knowledge on the efficacy of glucocorticoids, as well as the pharmacological principles, which should guide their optimal administration schedule in COVID-19.

## 2. The Role of Glucocorticoids in COVID-19

Glucocorticoids are probably the pharmacological treatment with the highest efficacy in COVID-19 pneumonia. The RECOVERY trial platform released its first preliminary findings in the spring of 2020, showing that the administration of 6 mg of dexamethasone for up to 10 days lowered the risk of death at 28 days (risk ratio (RR) 0.83; 95% CI 0.75–0.93), with a greater effect within the group of hospitalised patients who underwent mechanical ventilation (RR 0.64; 95% CI 0.51–0.81). In contrast, a higher risk of mortality was seen among subjects who received GCs and did not need respiratory assistance at randomization (RR 1.19; 95% CI 0.92–1.55) [16].

This first work was followed by many other RCTs (randomized controlled trials) and a large 2022 Cochrane meta-analysis, which included 9549 subjects from 16 RCTs, showing that GCs plus standard of care probably reduce all-cause death at 30 days compared to standard care alone (RR 0.90, 95% CI 0.84–0.97) [8]. Concordantly with the RECOVERY trial findings, mortality of patients receiving GCs was increased in the subgroup of milder patients who were not receiving oxygen therapy (RR 1.27; 95% CI 1.00–1.61), while it was more pronouncedly reduced in those requiring non-invasive ventilation (NIV) or high-flow nasal cannula (HFNC) compared to low-flow oxygen alone (RR 0.64; 95% CI 0.20–2.03 vs. 0.70; 95% CI 0.29–1.67). Furthermore, for those on GCs the likelihood of being discharged from the hospital alive at day 28 was slightly higher and the likelihood of needing invasive mechanical ventilation once again was slightly lower (RR 0.92, 95% CI 0.84–1.01). Comparing the GCs group to the control group, ventilator-free days rose by an average of 2.6 days. Notably, most patients (*n* = 3766) received dexamethasone. Finally, GCs tended to benefit more subjects aged <70 years than those aged >70 years, and the few individuals from a Black, Asian, or other minority ethnic group exhibited a bigger estimated impact than White participants.

Similarly to previous research on all-cause ARDS and severe COVID-19 [8,17], two RCTs [18,19] investigated and confirmed the efficacy of hydrocortisone (respectively, 150 mg daily for seven days and 200 mg daily for seven days, followed by tapering) in lowering the mortality risk (RR 0.78; 95% CI 0.57–1.05), which resulted to be even lower among the studies involving methylprednisolone (RR 0.63; 95% CI 0.26–1.54). Only one RCT, which investigated the efficacy of a lower dose and a shorter course (0.5 mg/Kg/day for five days) of methylprednisolone, resulted in non-superiority.

For what concerns the comparison of different doses of GCs, Taboada et al. [20]. randomly assigned 200 patients to receive lower-dose dexamethasone (6 mg once daily for 10 days) or higher-dose dexamethasone followed by tapering (20 mg once daily for five days, followed by 10 mg once daily for five additional days), reporting no statistically significant differences in clinical deterioration at 11 days, 28-day mortality, or any secondary endpoints. In another recent RCT, 1000 patients were randomly assigned to receive either 12 mg or 6 mg of dexamethasone once daily for 10 days in a 1:1 ratio. No statistically significant differences were found, but the 12 mg group showed a beneficial effect in terms of the number of days alive without support at 28 days [21]. Finally, according to a metanalysis of four RCTs, higher-dose dexamethasone seems to reduce all-cause mortality by 30 days compared to low-dose dexamethasone (RR 0.87, 95% CI 0.73–1.04) [4].

Methylprednisolone (MP) vs. dexamethasone (DM) effectiveness was investigated by a small number of RCTs. A single bolus of MP 2 mg/Kg/day for five days, then de-escalated to 1 mg/Kg/day for five days led to a statistically significant reduction in mortality vs. DM 6 mg/day for 10 days in 414 mechanically ventilated COVID-19 patients. Additionally, patients receiving MP experienced a greater decline in C-reactive protein (CRP) levels after 10 days and a shorter ICU (intensive care unit) stay (7.33 vs. 19.43 mean days) [22]. The same MP protocol failed to reach statistical significance in mortality reduction (RR 0.51; 95% CI 0.24–1.07) but resulted in a shorter hospital stay and a reduced need for mechanical ventilation (18.2% versus 38.1%) in another study on a smaller sample of 86 less severe patients [23]. This is not surprising, as there is substantial evidence of a proportional advantage of GCs among individuals who require mechanical ventilation (MV) compared with other lower-intensity respiratory support modalities [24].

An Italian RCT compared a bolus of IV methylprednisolone 80 mg, followed by a continuous intravenous infusion of 80 mg/day for eight days and then tapered over 14 days, to dexamethasone 6 mg/day for 10 days in 690 individuals receiving oxygen or noninvasive respiratory support due to COVID-19, finding no significant differences in mortality (10.4% vs. 12.1%) nor in median mechanical ventilation-free days [25]. A decreased risk of ICU admission and a noticeably larger reduction in CRP were seen on days seven and 14 among patients in the MP group with an arterial oxygen tension to a fraction of inspired oxygen ratio (PaO_2_:FiO_2_) <200 mmHg at randomization who completed the prescribed treatment [26]. There is evidence that persistently high CRP and the necessity for ICU admission are independent risk factors for the emergence of post-COVID conditions, and there is an association between a faster reduction of CRP and lower one-year mortality after sepsis and pneumonia [27,28].

Currently, all major recommendations call for the use of any GC compound equivalent to 6 mg of dexamethasone for seven to 10 days in moderate and severe COVID-19 [14]. In numerous randomized clinical trials, this therapeutic regimen has demonstrated safety and efficacy, lowering all-cause mortality without being linked to an increased risk of complications or unfavourable events. Despite its undeniable effectiveness, there is probably an opportunity for improvement. The next research efforts ought to concentrate on finding the optimal standard protocol as well as potential biomarkers that might help to personalize the dose and duration of therapy. Indeed, the substantial variations in GC plasma levels after administration of the same dose may be associated with a degree of resistance to GC and explain why COVID-19 may evolve severely in non-comorbid individuals as well [6].

## 3. Glucocorticoid Administration: Principles and Evidence

In a nutshell, glucocorticoids exert their effects by binding to the glucocorticoid receptor alpha (GRα). However, diverse substances have diverse pharmacological characteristics, and therapeutic effectiveness relies on the amount and length of exposure to glucocorticoids [6]. Based on data derived from studies on non-COVID19 ARDS, it is safe and logical to assume that the following principles, which were validated in the said studies on non-COVID19 ARDS, are likely to also provide the best outcomes in COVID-19 ARDS, due to the common pathogenetic pathway and similar clinical outcome shared from ARDS from all causes: an early intervention, a bolus dose to obtain close-to-maximum GRα saturation, followed by a continuous infusion to ensure high levels of response throughout the treatment period, then slow tapering to interruption [24]. Nonetheless, there is significant between-patient variability in plasma levels and intracellular response, which provides the rationale for modulating the dose and duration to achieve clinical and laboratory improvement [29].

### 3.1. Glucocorticoid Molecules

**Figure 1:**
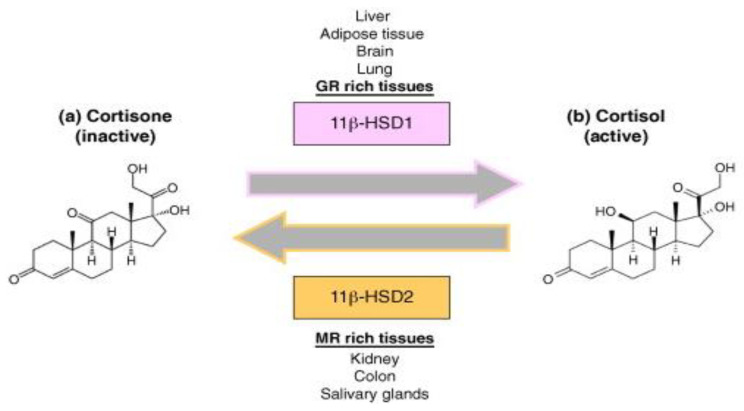
Glucocorticoid molecular mechanism. Legend: Molecular mechanism of 11β-HSD1 and 11β-HSD2 of converting cortisone (inactive) into its active form (cortisol) and vice versa.

Both the density of “nuclear” receptors and the pre-receptor intracellular metabolism by the two isozymes of 11 β-hydroxysteroid dehydrogenases type 1 and type 2 (11β-HSD1 and 11β-HSD2) have a role in determining the way glucocorticoids work on target tissues. By acting as an oxidoreductase (11β-reductase), 11β-HSD1 aids in the systemic regeneration of physiologically active glucocorticoids (cortisol/hydrocortisone, corticosterone, and prednisolone) from their inactive counterparts (respectively, cortisone, 11-dehydrocorticosterone, and prednisone). In contrast, 11β-HSD2 solely inactivates GCs and, interestingly, studies indicated insensitivity to glucocorticoids in cells that have a high 11β-HSD2 activity (see Figure 1) [30].

**Figure 2:**
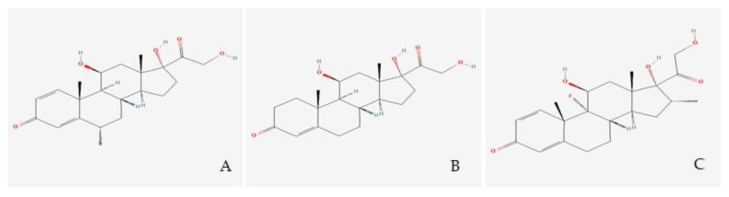
Glucocorticoid molecules. Legend. (**A**) Methylprednisolone; (**B**) Hydrocortisone; (**C**) Dexamethasone.

Dexamethasone and methylprednisolone, unlike hydrocortisone (see Figure 2), are not substrates to the 11β-HSD system, which makes their use preferable in the treatment of ARDS [31]. A post-mortem study performed on lung tissue from patients who suffered from ARDS, found high immunohistochemical expression of 11β-HSD2 [32], indicating a faster hydrocortisone breakdown, while dexamethasone (fluorinated in position 6a or 9a) and methylprednisolone (methylated at 2a or 6a) were not affected.

A549 cells, primary human lung epithelium (NHBE), and COVID-19 lung biopsies were used in a landmark COVID-19 transcriptomics study to examine the alterations in gene expression, pathways, and possible processes generated by SARS-CoV2 compared to controls. Thus, it was possible to identify 5694 FDA-approved medications that could be used to treat COVID-19 patients with severe symptoms of hyperinflammation. Among these, methylprednisolone had the greatest anti-inflammatory properties [33].

**Figure 3:**
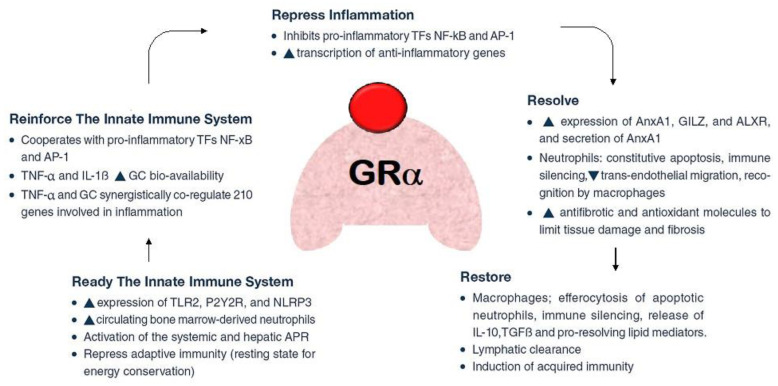
Pleiotropic function of glucocorticoid (in red colour) receptor-a (GRa, in pink colour). Legend: After acute damage, e.g., to the lung epithelium during acute respiratory distress syndrome (ARDS), GRa ensures the proper response through subsequent phases: (a) removal or neutralization of pathogens; (b) downregulation of inflammation to limit tissue damage; (c) restoration of tissue structure and function. These processes are associated with the upregulation and downregulation of multiple biochemical pathways, including switching production from pro-inflammatory to pro-resolving mediators.

GRβ, a natural dominant-negative inhibitor of hGRα-induced transactivation of glucocorticoid-responsive genes, is thought to be the cause of tissue-specific insensitivity to glucocorticoids, according to a number of clinically oriented studies [34]. Using experimental models, it was discovered that ARDS lung tissue expressed both more GRβ [35] and less GRα [35,36], consequently leading to a reduced nuclear translocation of GRα [35]. In contrast to hydrocortisone and dexamethasone, methylprednisolone was not influenced by the dominant negative effect of hGRβ, according to an in vitro analysis of the glucocorticoids examined in ARDS. The researchers noticed that both the type and the dose of synthetic glucocorticoids affect the dominant-negative effect of hGRβ on hGRα-induced transactivation [37] (Figure 3).

Using medications that have a high penetration rate into the damaged lung tissue to treat lung diseases may have therapeutic benefits. Although both methylprednisolone and dexamethasone were promptly absorbed into the lungs, methylprednisolone had a larger BALF exposure than prednisolone, according to two experimental trials that compared BALF levels of the two medicines to plasma levels. This difference was larger over time (longer residence time), implying that methylprednisolone remains in the BALF compartment for a longer time, i.e., it has a slower clearance from the lungs than from plasma [38,39]. Furthermore, continuous infusion granted a greater penetration compared to bolus administration [38].

### 3.2. Timing of Administration and Initial Dose

In order to lessen the acute and long-term detrimental effects of the allostatic load imposed during vital organ support, as emerged in various studies on critically ill patients requiring ventilation, early administration of GCs is essential, ideally within six hours of diagnosis [40,41,42,43,44,45]. A study performed in 2016 on ARDS proved that when fibroproliferation is still in the early stages of disease (cellular with a predominance of type III procollagen), early (72 h) as opposed to late (seven days) beginning of methylprednisolone administration is linked with quicker disease remission (extubation and ICU discharge) at the same dose (1 mg/kg/day vs. 2 mg/kg/day) [29].

**Figure 4:**
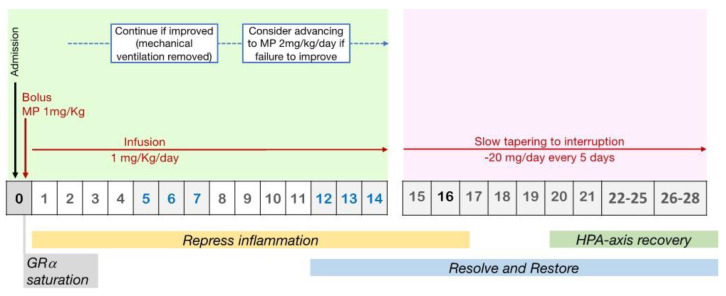
Protocol for prolonged methylprednisolone treatment in ARDS patients. Legend: Recommended protocol for prolonged methylprednisolone treatment in patients with early ARDS, involving an initial IV bolus to achieve rapid GR saturation, followed by an infusion to maintain high levels of response throughout the treatment period. GC treatment should be titrated on clinical worsening or amelioration and tapered to interruption.

From a pharmacological point of view, a loading bolus should always be administered to reach maximal glucocorticoid receptor-alpha (GRα) saturation (roughly 100 mg of methylprednisolone equivalent) in the cytoplasm and on the cell membrane, in order to overcome resistance imposed by increased GRβ [37,46]. Early ARDS patients should be administered 1 mg/kg/day of methylprednisolone, which is comparable to the dosage of dexamethasone (20 mg) used in the recent DEXA-ARDS randomized-controlled trial, which was performed just before the COVID-19 pandemic [47] and frequently prescribed for other interstitial lung disorders [48]. However, in some cases, such as (i) in the sickest patients with reduction adjustment based on FiO_2_ requirements and high CRP levels or (ii) in those on mechanical ventilation for five days or more (unresolving ARDS), a higher starting dose (i.e., methylprednisolone 2 mg/kg/day) may be necessary (Figure 4).

In-vitro research shed light on the effect of GC dose on inflammatory downregulation. The decrease of the transcription of inflammatory cytokine gene (TNF-α, IL-1β, and IL-6), regardless of baseline inflammation severity, was initially modest in human monocytic cells activated with lipopolysaccharide (LPS) and then exposed to increasing concentrations of methylprednisolone. It subsequently approached an inflexion point, which was followed by a swift decline, presumably as a result of getting near to attaining full drug receptor saturation for a noticeable genomic and nongenomic effect [49]. This discovery stresses the significance of selecting an appropriate dose to achieve GR saturation and the best outcomes.

### 3.3. Administration Modality

In terms of exposure, the typical GC plasma concentration-time profiles, represented as methylprednisolone equivalents, show significant variations between different GC regimens. The maintenance of GC effects in the target organs and tissues appears to be dependent on GC exposure in the target sites (e.g., plasma and the lungs). In fact, intervals of eight or 24 h between doses led to substantial periods with below-optimal GC serum exposure. On the other hand, continuous IV administration keeps plasma levels constantly high [6].

In various works on pharmacokinetics, severe pneumonia septic shock, it emerged that when compared to intermittent boluses, infusion allowed to achieve: (i) a lower variability in plasma concentration [50]; (ii) a better lung penetration; (iii) a steady, non-fluctuating exposure; (iv) higher response levels throughout administration [38]; (v) faster management of shock [51]; (vi) less nursing time for administration [52,53]; and (vii) fewer hyperglycemic episodes [54,55]. Recent research on COVID-19 ARDS seems to suggest that the implementation of this administration strategy, as opposed to intermittent bolusing, may lead to more rapid illness resolution, a greater number of days without mechanical ventilation (MV), and reduced hospital mortality [25].

### 3.4. Timing of GCs Administration and Tapering

The time frame of GC administration and tapering are key variables in determining therapy effectiveness. The aim of GC therapy in ARDS is to maintain the activated GRα central homeostatic function during the acute stages of the illness and the essential, but underestimated, resolution phase. Data from RCTs in COVID-19 showed that mortality is reduced with treatment durations of ten days or more [56].

According to critical care RCTs, abruptly withdrawing GC administration after three to 14 days of treatment (either intermittent boluses or continuous infusion) was quickly followed by a flared-up systemic inflammation with serious clinical relapses in about 33% of the patients [6], and this could be an indicator of greater mortality [57]. In particular, one-fourth of patients receiving methylprednisolone, in the LaSRS RCT [57], experienced a clinical rebound after discontinuing the study medicine within 36 h of successful extubation. These individuals did not receive GC therapy again because they needed to go back to MV. In contrast to patients who never went back to MV, this resulted in poor outcomes with extra days on MV and a nine-fold elevated risk of 60-day mortality (*p* = 0.001) [58] (Figure 4).

Case reports from the COVID-19 pandemic reveal similar events after stopping a ten-day dexamethasone treatment, with improvements following the reintroduction of GC medication [59,60].

### 3.5. Infection Surveillance and Clinical Monitoring during Mechanical Ventilation

In ARDS patients from all causes, the daily assessment of pulmonary function (lung injury score (LIS) [61] and respiratory rate) and multiple organ function (sequential organ failure assessment (SOFA) score] along with systemic inflammation markers (CRP and ferritin) is crucial for determining how well the therapy is working during the treatment course. While the rate of decline of inflammatory markers is directly proportional to the time required for the resolution of organ failure, persistently elevated inflammatory markers indicate an unfavourable outcome [62,63]. As a result of GCs administration, a swift lowering in inflammatory cytokine levels leads to a quick clinical improvement and vice versa [64,65].

The disparity in response amongst individuals with non-COVID19 ARDS undergoing a comparable treatment regimen may be attributed to the high interindividual heterogeneity in (i) plasma drug concentrations reached during therapy [50] and (ii) intracellular GRα sensitivity during GC treatment [66,67]. Notably, increasing GC dosages may compensate for reduced intracellular GRα sensitivity [66], indicating that intracellular GC resistance can be addressed with higher dosages (see Section 3.7). This explains why dose and duration modifications should be made tailored to clinical and laboratory responses.

Although it has not been demonstrated that extended GC therapy for ARDS increases the likelihood of superinfection, it is crucial to monitor infection signs during MV even in the absence of fever. In one RCT performed in 2007 on severe ARDS cases [68], meticulous protocol-based infection monitoring found 56% of nosocomial infections in patients who had no fever. In such context, procalcitonin, which is unaffected by GCs therapy (even methylprednisolone [69]), is a useful ally for early detection of bacterial superinfection [70,71,72,73]. A deteriorating LIS or SOFA score, an inexplicable increase in respiratory rate, a plateau or rise in CRP levels, and an increase in immature neutrophils are additional infection surveillance markers. In the absence of contraindications, monitoring weekly bronchoalveolar lavage (BAL) is useful for detecting ventilator-associated pneumonia and tracking lung inflammation (neutrophilia). Since most non-COVID19 ARDS patients who receive early prolonged GC treatment become ventilator-free within seven days of treatment, a weekly BAL for microbiological screening is not routinely indicated [29].

### 3.6. Post-Extubation Monitoring and Treatment Adjustment

Following weaning from MV, oxygen requirement offers a quick way to determine when lung function has fully recovered. It is recommendable to maintain GC treatment at a lower dose until the patient is able to breathe on room air, reaching satisfying oxygenation levels. After five days since MV cessation, transitioning to oral administration seems logical because one pharmacokinetic study (2005) found that intestine methylprednisolone absorption is impaired for approximately five days after extubation only [50].

### 3.7. Independent Factors Affecting Response to GCs Treatment

As reported in Table 1, factors connected to (i) the patient’s comorbidities and (ii) the critical illness itself can have an impact on a patient’s reaction to endogenous glucocorticoids in critically ill and ARDS patients. Comorbidities linked to GC resistance [74,75], cellular sensitivity [67,76], and less frequent GR polymorphisms [77] are patient-related factors. On the other hand, critical disease-related factors, include (i) pre-receptor metabolism by 11β-HSD1 and 11β-HSD2 (ii), GR number and isoforms (a vs. b) [69,78,79,80,81,82]- as emerged in a study on COVID-19 itself [82]-, (iii) decreased HDL cholesterol levels [83] and intracellular penetration [84,85,86,87,88,89,90,91,92], (iv) impact of micronutrient deficiency on GC availability and GC-GR function [85,87,88,89,90,91,92], (v) impact of inflammatory state (induce non-compensated GC resistance in target organs) [93,94,95] and oxidative stress [85,96,97,98,99,100]. Most of these factors may also influence how well a patient responds to glucocorticoid therapy (Table 2).

Inter-individual variability in response to GC administration remains an unpredictable and uncontrollable variable factor. Indeed, it is still unknown whether glucocorticoid resistance in critically ill patients is a primary phenomenon, or whether the anti-inflammatory property of glucocorticoids is simply underpowered to face the overexpression of pro-inflammatory cytokines [101]. The significant heterogeneity in (i) reachable plasma drug concentrations [50] and (ii) intracellular glucocorticoid receptor sensitivity during GC treatment [66,67] has been brought to light by three investigations, performed respectively on young healthy patients, patients with septic shock abdARDS patients. In the first one, GC sensitivity was assessed by exposing concanavalin A-stimulated lymphocytes of 40 healthy volunteers to increasing concentrations of dexamethasone. At every tested dose, significant variation in the inhibition of lymphocyte proliferation was observed [66]. Furthermore, the effect increased with increasing GC doses, indicating that higher doses can overcome intracellular GC resistance [63]. Another similar research found a significant intra-individual variation among healthy volunteers [102,103]. In the second research, which involved individuals who had had septic shock within three days, GC sensitivity was evaluated by inhibiting the ability of leukocytes to produce cytokines in vitro [67]. The sensitivity to DM increased in many patients, but some others have shown an opposite pattern. In particular, patients with a lower GC sensitivity had more severe disease, as shown by higher APACHE II scores [67]. Notably, GRβ or 11β-HSD-2 mRNA expression was not higher among patients in the lowest quartile of glucocorticoid sensitivity, consistent with previous research and suggesting that the large variance in lymphocyte suppression sensitivity has a post-receptor basis [102]. The third investigation [68] is a pharmacokinetic one that analysed the concentration-time profiles of methylprednisolone in 20 ARDS patients who were administered methylprednisolone 1 mg/kg ideal body weight (IBW) within an RCT, finding significant heterogeneity in plasma concentrations. The average methylprednisolone level was 203 ± 127 ng/mL at the steady state during continuous infusion, and the range was 50–820 ng/mL. Variability, which reached as high as a 10-fold, was larger after bolus and lower with continuous infusion [54]. There was no distinction in glucocorticoid resistance between males and females [66,102].

## 4. Complications and Adverse Events

The prolonged administration of GCs can lead to consequences affecting gastrointestinal, cardiovascular, endocrine, nervous, ocular, and immune systems. However, this is less likely to occur with the short courses of GC used in critical care than with the longer ones which are required, for example, in rheumatological diseases [104]. Therefore, the benefit/risk ratio is favourable in ARDS, including SARS-CoV-2-related ARDS. Possible complications arising during GC treatment have been examined in hundreds of patients with severe sepsis, septic shock, ARDS, and COVID-19.

A 2017 Cochrane meta-analysis showed an increase in cases of hyperglycemia (RR 1.72; 95% CI 1.38–2.14) linked to the use of GCs [105]; beyond this, the numerous comparisons between people treated with GCs and controls, also performed during the COVID-19 pandemic, did not show significant differences in the incidence of secondary infections or adverse events (RR 1.19; 95% CI 0.73–1.93). In the studies, hyperglycemia occurred without other significant consequences and was self-resolved after suspension of GC treatment [19,26,106,107,108]. Only one early study reported more superinfections and bleeding in patients treated with hydrocortisone compared to placebo [18]. Three RCTs reported an increased incidence of hospital-acquired superinfections: of these, two studies were underpowered [107,109] and one involved the use of high-dose, pulsed, methylprednisolone [106]. Although not confirmed by RCTs and multivariate models, some studies have shown an increased incidence of pulmonary aspergillosis (CAPA) in subjects treated for 10 days with 6 mg of dexamethasone per day [110]. Low-dose GC therapy did not delay viral shedding and there is currently no scientific evidence establishing a link between viral shedding and outcome in critically ill COVID-19 patients [26,111].

Adrenal insufficiency has been reported in 30% of patients treated with long-term GCs [112]. Indeed, exogenous GC therapy inhibits the HPA axis, causing negative feedback on endogenous GCs production, which is restored more slowly by the adrenal gland once drug treatment is stopped. While this is not a concern for short courses of GCs, studies showed a greater risk of returning to mechanical ventilation and an increase in inflammation indices if treatment is abruptly stopped [113]. A gradual reduction of GCs is therefore recommended, slowly tapering the treatment to interruption.

## 5. Advantages and Disadvantages of Other Standardized Protocols Using GC in COVID-19

Once glucocorticoids were validated by numerous studies and international guidelines, the attention of the scientific community focused on the use of additional therapeutic protocols for the treatment of COVID-19. Regarding severe SARS-CoV-2 infection forms, Tocilizumab, Baricitinib, Anakinra and Remdesevir are approved for use in combination with the standard of care that includes steroids [114,115].

Tocilizumab is a recombinant humanised anti-IL-6 receptor monoclonal antibody that inhibits the binding of IL-6 to both membrane and soluble IL-6 receptors, key mediator in the inflammatory response during COVID-19 [116].

Several studies compared Tocilizumab plus standard of care versus standard of care alone, in particular, RECOVERY is the largest randomised, controlled, open-label clinical trial that considered patients on steroid therapy alone versus patients on Tocilizumab therapy, with or without the use of glucocorticoids. The RECOVERY study group confirmed that the benefit of Tocilizumab therapy was more consistent in the group of patients on combination steroid therapy, particularly in terms of 28-day mortality, but also on other clinical outcomes such as hospital discharge and disease progression [117]. From these results, together with those of other large-scale work, both the US Food and Drug Administration (FDA) and European Medicines Agency (EMA) approved the use of Tocilizumab in hospitalised adults receiving systemic corticosteroids and requiring oxygen, NIV, IVM, or ECMO [118,119]. Based on the same concept of host immune response-based therapy, the use of anakinra, an anti-IL-1 monoclonal antibody directed against another of the key cytokines in the development of severe SARS-CoV-2 disease, was confirmed. The use of anakinra, both alone and together with steroid therapy, demonstrated both a reduction in 28-day mortality and the need for non-invasive mechanical ventilation. This drug is therefore also approved by both the EMA and FDA for use in patients with severe forms of SARS-CoV-2 [120]. The clear role of the Janus kinase/signal transducer and activator of transcription proteins (JAK/STAT) pathway in the cytokine storm typical of severe forms of COVID-19 has led to the use of JAK/STAT inhibitors, including baricitinib. Again, the protocol of use analysed in most studies was combination therapy with JAK/STAT versus standard of care. RECOVERY performed a randomised, controlled, open-label, platform trial with the largest number of patients in the literature to date, confirming the effect of Baricitinib on mortality reduction. This drug is currently FDA licensed in the US but has not yet received approval in Europe [121].

Remdesivir is an antiviral medicine approved for the treatment of mild-to-moderate SARS-CoV-2 infections. A recent Cochrane meta-analysis analysed the body of literature available up to May 2022 on the use of remdesevir, alone or in combination with standard of care, showing little or no effect on all-cause mortality or in-hospital mortality of individuals with moderate to severe COVID-19 [122]. Despite this, several studies confirmed that combination therapy with different glucocorticoid molecules (such as dexamethosone or methylprednisolone) and remdesevir could have some effects on survival in patients with moderate or severe forms of COVID-19 [123,124,125]. In any case, the use of Remdesevir is currently approved by both the FDA and EMA for adults and pediatric patients hospitalized or not hospitalized, with mild-to-moderate SARS-CoV-2 infections and at high risk for progression to severe forms [118,119].

Other drugs have been tested, either alone or in combination with glucocorticoids, for the treatment of SARS-CoV-2 infection, including interferon and granulocyte–macrophage colony-stimulating factor (Anti-GM-CSF) but none of these is currently included in the guidelines. However, there is no therapy to date that is superior to the use of steroids, but several treatment protocols are proposed as combination therapy and have shown improved patient outcomes [114].

## 6. Conclusions

Glucocorticoids use in ARDS from severe SARS-CoV-2 infection has been extensively validated by several studies and is fully included in international guidelines. The use of low doses of cortisteroids is actually approved and recommended for the early-stage treatment of ARDS in adults [126]. In particular, the use of low doses of glucocorticoids is able to control, at least in part, the non-specific inflammation that characterizes the ARDS and, in practice, reduce mortality and the duration of mechanical ventilation [127]. In fact, the use of steroids in ARDS appears to have different effects on patients’ outcomes depending on the cause, if any, which led to the onset of the condition [128]. For several years, the use of steroids has been proposed, with increasing approval, for the treatment of severe community-acquired pneumonia (CAP), again based on the rationale of controlling and modulating inflammation [129,130,131,132,133,134]. With the knowledge available to date, in general, the use of steroid therapy in ARDS from any cause should be assessed on the basis of the patient’s clinical features and the aetiology of the respiratory insufficiency, taking into account that the benefit of therapy depends on several factors and the risks, particularly infectious ones, associated with the use of these drugs [129,130,131,132,133,134]. As far as severe SARS-CoV-2 infections are concerned, the use of glucocorticoids is currently unquestionably validated. Future prospects should focus on which molecule, if any, is the best and which clinical, laboratory and radiological patient characteristics can guide towards a tailored therapy, also in combination with the other immunomodulatory drugs available today.

## Figures and Tables

**Table 1:** Summary of the main characteristics and findings of the works included in paragraph 2.

Authors and Year of Publication	Title	Design	Drugs	Examined Patients	Results
The RECOVERY Collaborative Group (2021) [16]	Dexamethasone in hospitalized patients with COVID-19	Prospective randomised trial compared with placebo	Dexamethasone 6 mg vs. placebo	4321 placebo patients vs. 2104 treated, hospitalised with COVID-19 confirmed	Only patients needing oxygen support showed lower mortality
Wagner C, Griesel M, Mikolajewska A, et al. (2021) [8]	Systemic corticosteroids for the treatment of COVID-19.	Living systematic review	A total of 3072 participants were randomised to corticosteroid arms and the majority received dexamethasone (*n* = 2322).	11 RCTs in 8075 participants, of whom 7041 (87%) originated from high-income countries.	11 studies with 8075 people. About 3000 people received corticosteroids, mostly dexamethasone (2322 people). Most studies took place in high-income countries. There were also found 42 ongoing studies, and 16 completed studies that have not yet published their results.
The COVID STEROID 2 Trial Group (2021) [21]	Effect of 12 mg vs. 6 mg of dexamethasone on the number of days alive without life support in adults with COVID-19 and severe hypoxemia: The COVID STEROID 2 randomized trial	Multicentre, randomised clinical trial	IV Dexamethasone 12 mg vs. IV Dexamethasone 6 mg IV	982 adult patients with COVID-19 needing at least 10 L/min of oxygen or MV (mechanical ventilation)	No statistically significant difference on ventilator-free days over 28 days
The Writing Committee for the REMAP-CAP Investigators, Angus DC, Derde L, Al-Beidh F, Annane D, Arabi Y, et al. (2020) [18]	Effect of hydrocortisone on mortality and organ support in patients with severe COVID-19: The REMAP-CAP COVID-19 corticosteroid domain randomized clinical trial	Bayesian randomised clinical trial	No hydrocortisone vs. hydrocortisone (50 mg or 100 mg every 6 h or shock-dependent dosage)	108 non-treated vs. 295 treated adult patients in ICU with severe SARS-CoV-2 pneumonia	Patients treated with hydrocortisone showed improvement in organ support-free days within 21 days
Dequin P-F, Heming N, Meziani F, Plantefève G, Voiriot G, Badié J, et al. (2020) [19]	Effect of hydrocortisone on 21-day mortality or respiratory support among critically ill patients with COVID-19: A randomized clinical trial	Multicentre randomised double-blind sequential trial	Hydrocortisone 200 mg/die tapered to 100 mg and then 50 mg vs. placebo	73 placebo vs. 76 treated, patients admitted to ICU with ARDS secondary to COVID-19 infection	The study was stopped early due to no significant reduction in treatment failure in hydrocortisone group
Sterne JAC, Murthy S, Diaz J V., et al. (2020) [4]	Association Between Administration of Systemic Corticosteroids and Mortality Among Critically Ill Patients With COVID-19.	Prospective meta-analysis	Systemic hydrocortisone, dexamethasone, or methylprednisolone (678 patients) or usual care or placebo (1025 patients).	1703 patients with critical COVID-19 from 7 RCTs	Administration of systemic corticosteroids, compared with usual care or placebo, was associated with lower 28-day all-cause mortality
Salton F, Confalonieri P, Meduri GU, Santus P, Harari S, Scala R, et al. (2020) [26]	Prolonged low-dose methylprednisolone in patients with severe COVID-19 pneumonia	Multicentre, observational, longitudinal study	Methylprednisolone 80 mg IV, followed by an infusion of 80 mg/d in 240 mL of normal saline at 10 mL/h for at least 8 days, until achieving either a PaO_2_:FiO_2_ > 350 mmHg or a CRP < 20 mg/L; then MP 16 mg o.so or 20 mg IV twice daily until CRP reached < 20% of the normal range or a PaO_2_:FiO_2_ > 400 (alternative SatO_2_ ≥ 95% in room air)	83 treated patients vs. 90 control patients with severe COVID-19 pneumonia	Early administration of prolonged, low dose MP treatment was associated with a significantly lower hazard of death and decreased ventilator dependence
Ranjbar K, Moghadami M, Mirahmadizadeh A, Fallahi MJ, Khaloo V, Shahriarirad R, et al. (2021) [23]	Methylprednisolone or dexamethasone, which one is superior corticosteroid in the treatment of hospitalized COVID-19 patients: A triple-blinded randomized controlled trial	Prospective triple-blinded randomised controlled trial	Methylprednisolone (2 mg/kg/day) or dexamethasone (6 mg/day)	47 patients on MP vs. 46 patients on DM hospitalised with COVID-19 pneumonia	Superiority of methylprednisolone over dexamethasone
Saeed MAM, Mohamed AH, Owaynat AH. (2022) [22]	Comparison between methylprednisolone infusion and dexamethasone in COVID-19 ARDS mechanically ventilated patients	Prospective cohort study	Dexamethasone 6 mg/day vs methylprednisolone 2 mg/kg/day IV	192 patients treated with dexamethasone vs. 222 patients treated with MP admitted in ICU with SARS-CoV-2 pneumonia (confirmed)	The methylprednisolone group showed an improvement of the inflammatory markers for cytokine storm in comparison to the patients on dexamethasone
Salton F, Confalonieri P, Centanni S, Mondoni M, Petrosillo N, Bonfanti P, et al. (2022) [25]	Prolonged higher dose methylprednisolone vs. conventional dexamethasone in COVID-19 pneumonia: A randomised controlled trial (MEDEAS)	Multicentre, open-label randomised clinical trial	Methylprednisolone 80 mg IV in continuous daily infusion for 8 days followed by slow tapering vs. dexamethasone 6 mg daily	337 patients treated with methylprednisolone vs. 340 in dexamethasone group with COVID-19 pneumonia requiring oxygen or non-invasive respiratory support	No significant differences in mortality between the two groups
Taboada M, Rodríguez N, Varela PM, et al. (2022) [20]	Effect of high *versus* low dose of dexamethasone on clinical worsening in patients hospitalised with moderate or severe COVID-19 pneumonia: an open-label, randomised clinical trial.	Randomised, open-label, controlled trial	Patients were randomly assigned in a 1:1 ratio to receive low-dose dexamethasone (6 mg once daily for 10 days) or high-dose dexamethasone (20 mg once daily for 5 days, followed by 10 mg once daily for an additional 5 days).	200 hospitalised patients with confirmed COVID-19 pneumonia needing oxygen therapy	Among hospitalised COVID-19 patients needing oxygen therapy, high dose of dexamethasone reduced clinical worsening within 11 days after randomisation, compared with low dose.
Lamontagne F, Agarwal A, Rochwerg B, et al. (2020) [14]	A living WHO guideline on drugs for COVID-19	Living guideline based on living systematic review and network analysis	Remdesivir 200 mg IV on the first day then 100 mg IV/day for 5–10 days and corticosteroids (dexamethasone 6 mg oral or IV once daily for 7–10 days, hydrocortisone 50 mg IV every 8 h for 7–10 days, methylprednisolone 10 mg IV every 6 h for 7–10 days, prednisone 40 mg oral daily for 7–10 days)	For remdesivir 4 studies with a total of 7347 patients ranging from non-severe to critical; for corticosteroids 8 RCTs with a total of 7184 patients ranging from non-severe to critical	Recommendation against remdesivir (weak); Recommendation in favour of corticosteroids (suggested regimen dexamethasone 6 mg oral or IV once daily for 7–10 days) for patients with severe and critical COVID-19 (strong), recommendation against corticosteroids in patients with non-severe COVID-19 (weak)
Hirano Y, Madokoro S, Kondo Y, Okamoto K, Tanaka H [17].	Corticosteroid treatment for early acute respiratory distress syndrome: a systematic review and meta-analysis of randomized trials	Systematic review and meta-analysis of randomized controlled trials (RCTs)	RCTs analysing the efficacy of prolonged corticosteroid (methylprednisolone, dexamethasone) therapy in early ARDS	4 RCTs, 385 patients in the corticosteroid group, 357 in the control group	Prolonged corticosteroid treatment in early ARDS improved the survival outcomes.

**Table 2:** Factors affecting response to glucocorticoid treatment.

**Disease-Related Factors**	**GR Number and Type (α vs. β)**	
	**GR Binding Capacity (Bmax)**	**▼ by Inflammatory State**
		**▼ by Oxidative Stress**
	**GR Function**	▼ by Hypovitaminosis *, Micronutrient Deficiencies
**Molecule-related factors**	Binding affinity to GR (potency)	Increasing the dosage of GC can compensate for a reduced binding affinity
**Treatment-related factors**	initiation	GR saturation (dose-dependent)
		Timing of initiation (early vs. late)
		Dose-adjustments based on initial severity ‡
	over time	Optimal magnitude of exposure to GR → Bolus followed by continuous infusion
		Lung penetration and tissue concentration
		Dose-adjustments based on clinical response §
		Duration of treatment including tapering
	potential complications	Hypothalamic-pituitary-axis suppression → Can be offset with slow tapering
		Failure to recognize infections in absence of fever → Can be offset with infection surveillance
**Patients-related factors**	Comorbidities associated with GC resistance †	
	Interpersonal wide variability in achieved GC plasma concentration	
	Interpersonal wide variability in cellular sensitivity to exposure of similar GC concentration	

**Legend**: ▼, decreased; GR, glucocorticoid receptor; Bmax, total density (concentration) of receptors in a sample of tissue. * Hypovitaminosis, three vitamins, thiamine (vitamin B1), ascorbic acid (vitamin C), and vitamin D, are important for the proper functioning of the GR system and mitochondria (but their reserves are rapidly exhausted in critical illness; † Comorbidities associated with GC resistance: obesity, chronic lung, and heart diseases, smocking, etc. [74,75]. ‡ A larger initial dose (i.e., methylprednisolone 2 mg/kg/day) may be required in patients requiring high oxygen levels (FiO_2_ ≥ 0.8); § adjustment based on C-reactive protein levels and PaO_2_:FiO_2_.

## Data Availability

Not applicable.

## References

[1] Rhen T., Cidlowski J.A. (2005). Antiinflammatory Action of Glucocorticoids—New Mechanisms for Old Drugs. N. Engl. J. Med..

[2] Bruscoli S., Puzzovio P.G., Zaimi M., Tiligada K., Levi-Schaffer F., Riccardi C. (2022). Glucocorticoids and COVID-19. Pharmacol. Res..

[3] Annane D., Pastores S.M., Rochwerg B., Arlt W., Balk R.A., Beishuizen A., Briegel J., Carcillo J., Christ-Crain M., Cooper M.S. (2017). Guidelines for the Diagnosis and Management of Critical Illness-Related Corticosteroid Insufficiency (CIRCI) in Critically Ill Patients (Part I). Crit. Care Med..

[4] Sterne J.A.C., Murthy S., Diaz J.V., Slutsky A.S., Villar J., Angus D.C., Annane D., Azevedo L.C.P., Berwanger O., Cavalcanti A.B. (2020). Association Between Administration of Systemic Corticosteroids and Mortality Among Critically Ill Patients with COVID-19. JAMA.

[5] Siemieniuk R.A.C., Meade M.O., Alonso-Coello P., Briel M., Evaniew N., Prasad M., Alexander P.E., Fei Y., Vandvik P.O., Loeb M. (2015). Corticosteroid Therapy for Patients Hospitalized with Community-Acquired Pneumonia. Ann. Intern. Med..

[6] Meduri G.U., Annane D., Confalonieri M., Chrousos G.P., Rochwerg B., Busby A., Ruaro B., Meibohm B. (2020). Pharmacological principles guiding prolonged glucocorticoid treatment in ARDS. Intensive Care Med..

[7] Pastores S.M., Annane D., Rochwerg B. (2018). Guidelines for the diagnosis and management of critical illness-related corticosteroid insufficiency (CIRCI) in critically ill patients (Part II): Society of Critical Care Medicine (SCCM) and European Society of Intensive Care Medicine (ESICM). Intensive Care Med..

[8] Wagner C., Griesel M., Mikolajewska A., Mueller A., Nothacker M., Kley K., Metzendorf M.I., Fischer A.L., Kopp M., Stegemann M. (2021). Systemic corticosteroids for the treatment of COVID. Cochrane Database Syst. Rev..

[9] Yang Z., Liu J., Zhou Y., Zhao X., Zhao Q., Liu J. (2020). The effect of corticosteroid treatment on patients with coronavirus infection: A systematic review and meta-analysis. J. Infect..

[10] Arabi Y.M., Mandourah Y., Al-Hameed F., Sindi A.A., Almekhlafi G.A., Hussein M.A., Jose J., Pinto R., Al-Omari A., Kharaba A. (2018). Corticosteroid Therapy for Critically Ill Patients with Middle East Respiratory Syndrome. Am. J. Respir. Crit. Care Med..

[11] Ogimi C., Greninger A.L., Waghmare A.A., Kuypers J.M., Shean R.C., Xie H., Leisenring W.M., Stevens-Ayers T.L., Jerome K.R., Englund J.A. (2017). Prolonged Shedding of Human Coronavirus in Hematopoietic Cell Transplant Recipients: Risk Factors and Viral Genome Evolution. J. Infect. Dis..

[12] Cao B., Gao H., Zhou B., Deng X., Hu C., Deng C., Lu H., Li Y., Gan J., Liu J. (2016). Adjuvant Corticosteroid Treatment in Adults with Influenza A (H7N9) Viral Pneumonia. Crit. Care Med..

[13] Boudreault A.A., Xie H., Leisenring W., Englund J., Corey L., Boeckh M. (2011). Impact of Corticosteroid Treatment and Antiviral Therapy on Clinical Outcomes in Hematopoietic Cell Transplant Patients Infected with Influenza Virus. Biol. Blood Marrow Transplant..

[14] Lamontagne F., Agarwal A., Rochwerg B., Siemieniuk R.A., Agoritsas T., Askie L., Lytvyn L., Leo Y.S., Macdonald H., Zeng L. (2020). A living WHO guideline on drugs for COVID-19. BMJ.

[15] Bone R.C., Fisher C.J., Clemmer T.P., Slotman G.J., Metz C.A., Balk R.A. (1987). A Controlled Clinical Trial of High-Dose Methylprednisolone in the Treatment of Severe Sepsis and Septic Shock. N. Engl. J. Med..

[16] The RECOVERY Collaborative Group (2021). Dexamethasone in Hospitalized Patients with COVID-19. N. Engl. J. Med..

[17] Hirano Y., Madokoro S., Kondo Y., Okamoto K., Tanaka H. (2020). Corticosteroid treatment for early acute respiratory distress syndrome: A systematic review and meta-analysis of randomized trials. J. Intensive Care.

[18] Angus D.C., Derde L., Al-Beidh F., Annane D., Arabi Y., Beane A., van Bentum-Puijk W., Berry L., Bhimani Z., Bonten M. (2020). Effect of Hydrocortisone on Mortality and Organ Support in Patients with Severe COVID-19. JAMA.

[19] Dequin P.F., Heming N., Meziani F., Plantefève G., Voiriot G., Badié J., François B., Aubron C., Ricard J.D., Ehrmann S. (2020). Effect of Hydrocortisone on 21-Day Mortality or Respiratory Support Among Critically Ill Patients with COVID-19. JAMA.

[20] Taboada M., Rodríguez N., Varela P.M., Rodríguez M.T., Abelleira R., González A., Casal A., Díaz Peromingo J.A., Lama A., Domínguez M.J. (2022). Effect of high versus low dose of dexamethasone on clinical worsening in patients hospitalised with moderate or severe COVID-19 pneumonia: An open-label, randomised clinical trial. Eur. Respir. J..

[21] Maskin L.P., Bonelli I., Olarte G.L., Palizas F., Velo A.E., Lurbet M.F., Lovazzano P., Kotsias S., Attie S., Lopez Saubidet I. (2022). High- Versus Low-Dose Dexamethasone for the Treatment of COVID-19-Related Acute Respiratory Distress Syndrome: A Multicenter, Randomized Open-Label Clinical Trial. J. Intensive Care Med..

[22] Saeed M.A.M., Mohamed A.H., Owaynat A.H. (2022). Comparison between methylprednisolone infusion and dexamethasone in COVID-19 ARDS mechanically ventilated patients. Egypt. J. Intern. Med..

[23] Ranjbar K., Moghadami M., Mirahmadizadeh A., Fallahi M.J., Khaloo V., Shahriarirad R., Erfani A., Khodamoradi Z., Gholampoor Saadi M.H. (2021). Methylprednisolone or dexamethasone, which one is superior corticosteroid in the treatment of hospitalized COVID-19 patients: A triple-blinded randomized controlled trial. BMC Infect. Dis..

[24] Hsieh Y.H., Chang H.T., Wang P.H., Chang M.Y., Hsu H.S. (2023). Mortality in patients with COVID-19 versus non-COVID-19- related acute respiratory distress syndrome: A single center retrospective observational cohort study. PLoS ONE.

[25] Salton F., Confalonieri P., Centanni S., Mondoni M., Petrosillo N., Bonfanti P., Lapadula G., Lacedonia D., Voza A., Carpenè N. (2023). Prolonged higher dose methylprednisolone *versus* conventional dexamethasone in COVID-19 pneumonia: A randomised controlled trial (MEDEAS). Eur. Respir. J..

[26] Salton F., Confalonieri P., Meduri G.U., Santus P., Harari S., Scala R., Lanini S., Vertui V., Oggionni T., Caminati A. (2020). Prolonged Low-Dose Methylprednisolone in Patients with Severe COVID-19 Pneumonia. Open Forum Infect. Dis..

[27] Maamar M., Artime A., Pariente E., Fierro P., Ruiz Y., Gutiérrez S., Tobalina M., Díaz-Salazar S., Ramos C., Olmos J.M. (2022). Post-COVID-19 syndrome, low-grade inflammation and inflammatory markers: A cross-sectional study. Curr. Med. Res. Opin..

[28] Mandal S., Barnett J., Brill S.E., Brown J.S., Denneny E.K., Hare S.S., Heightman M., Hillman T.E., Jacob J., Jarvis H.C. (2021). ‘Long-COVID’: A cross-sectional study of persisting symptoms, biomarker and imaging abnormalities following hospitalisation for COVID-19. Thorax.

[29] Meduri G.U., Bridges L., Shih M.C., Marik P.E., Siemieniuk R.A.C., Kocak M. (2016). Prolonged glucocorticoid treatment is associated with improved ARDS outcomes: Analysis of individual patients’ data from four randomized trials and trial-level meta-analysis of the updated literature. Intensive Care Med..

[30] Schreiber S.N., Emter R., Hock M.B., Knutti D., Cardenas J., Podvinec M., Oakeley E.J., Kralli A. (2004). The estrogen-related receptor α (ERRα) functions in PPARγ coactivator 1α (PGC-1α)-induced mitochondrial biogenesis. Proc. Natl. Acad. Sci. USA.

[31] Diederich S., Eigendorff E., Burkhardt P., Quinkler M., Bumke-Vogt C., Rochel M., Seidelmann D., Esperling P., Oelkers W., Bähr V. (2002). 11β-Hydroxysteroid Dehydrogenase Types 1 and 2: An Important Pharmacokinetic Determinant for the Activity of Synthetic Mineralo- and Glucocorticoids. J. Clin. Endocrinol. Metab..

[32] Suzuki S., Tsubochi H., Ishibashi H., Suzuki T., Kondo T., Sasano H. (2003). Increased expression of 11beta-hydroxysteroid dehydrogenase type 2 in the lungs of patients with acute respiratory distress syndrome. Pathol. Int..

[33] Draghici S., Nguyen T.M., Sonna L.A., Ziraldo C., Vanciu R., Fadel R., Morrison A., Kenney R.M., Alangaden G., Ramesh M. (2021). COVID-19: Disease pathways and gene expression changes predict methylprednisolone can improve outcome in severe cases. Bioinformatics.

[34] Kino T., Su Y.A., Chrousos G.P. (2009). Human glucocorticoid receptor isoform β: Recent understanding of its potential implications in physiology and pathophysiology. Cell. Mol. Life Sci..

[35] Kamiyama K., Matsuda N., Yamamoto S., Takano K., Takano Y., Yamazaki H., Kageyama S., Yokoo H., Nagata T., Hatakeyama N. (2008). Modulation of glucocorticoid receptor expression, inflammation, and cell apoptosis in septic guinea pig lungs using methylprednisolone. Am. J. Physiol. Lung Cell. Mol. Physiol..

[36] Wang X.Q., Zhou X., Zhou Y., Rong L., Gao L., Xu W. (2008). Low-dose dexamethasone alleviates lipopolysaccharide-induced acute lung injury in rats and upregulates pulmonary glucocorticoid receptors. Respirology.

[37] Fruchter O., Kino T., Zoumakis E., Alesci S., De Martino M., Chrousos G., Hochberg Z. (2005). The Human Glucocorticoid Receptor (GR) Isoform β Differentially Suppresses GRα-Induced Transactivation Stimulated by Synthetic Glucocorticoids. J. Clin. Endocrinol. Metab..

[38] Vichyanond P., Irvin C., Larsen G., Szefler S., Hill M. (1989). Penetration of corticosteroids into the lung: Evidence for a difference between methylprednisolone and prednisolone. J. Allergy Clin. Immunol..

[39] Greos L.S., Vichyanond P., Bloedow D.C., Irvin C.G., Larsen G.L., Szefler S.J., Hill M.R. (1991). Methylprednisolone Achieves Greater Concentrations in the Lung Than Prednisolone: A Pharmacokinetic Analysis. Am. Rev. Respir. Dis..

[40] Meduri G.U., Chrousos G.P. (2020). General Adaptation in Critical Illness: Glucocorticoid Receptor-alpha Master Regulator of Homeostatic Corrections. Front. Endocrinol..

[41] Combes A., Costa M.A., Trouillet J.L., Robinson C., Sganzerla D., Loss S.H., Lora P., Micheletti V.D. (2003). Morbidity, mortality, and quality-of-life outcomes of patients requiring ≥14 days of mechanical ventilation. Crit. Care Med..

[42] Wilson M.E., Barwise A., Heise K.J., Loftsgard T.O., Dziadzko M., Cheville A., Majzoub A., Novotny P.J., Gajic O., Biehl M. (2018). Long-Term Return to Functional Baseline After Mechanical Ventilation in the ICU. Crit. Care Med..

[43] Zilberberg M.D., Nathanson B.H., Ways J., Shorr A.F. (2020). Characteristics, Hospital Course, and Outcomes of Patients Requiring Prolonged Acute Versus Short-Term Mechanical Ventilation in the United States, 2014–2018. Crit. Care Med..

[44] Giordano G., Pugliese F., Bilotta F. (2020). Mechanical ventilation and long-term neurocognitive impairment after acute respiratory distress syndrome. Crit. Care.

[45] Straub R.H., Schradin C. (2016). Chronic inflammatory systemic diseases—An evolutionary trade-off between acutely beneficial but chronically harmful programs. Evol. Med. Public Health.

[46] Buttgereit F. (2002). Standardised nomenclature for glucocorticoid dosages and glucocorticoid treatment regimens: Current questions and tentative answers in rheumatology. Ann. Rheum. Dis..

[47] Villar J., Ferrando C., Martínez D., Ambrós A., Muñoz T., Soler J.A., Aguilar G., Alba F., González-Higueras E., Conesa L.A. (2020). Dexamethasone treatment for the acute respiratory distress syndrome: A multicentre, randomised controlled trial. Lancet Respir. Med..

[48] Jantz M.A., Sahn S.A. (1999). Corticosteroids in Acute Respiratory Failure. Am. J. Respir. Crit. Care Med..

[49] Meduri G.U., Kanangat S., Bronze M., Patterson D.R., Meduri C.U., Pak C., Tolley E.A., Schaberg D.R. (2001). Effects of Methylprednisolone on Intracellular Bacterial Growth. Clin. Diagn. Lab. Immunol..

[50] Yates C.R., Vysokanov A., Mukherjee A., Ludden T.M., Tolley E., Meduri G.U., Dalton J.T. (2001). Time-Variant Increase in Methylprednisolone Clearance in Patients with Acute Respiratory Distress Syndrome: A Population Pharmacokinetic Study. J. Clin. Pharmacol..

[51] Ibarra-Estrada M.A., Chávez-Peña Q., Reynoso-Estrella C.I., Rios-Zermeño J., Aguilera-González P.E., García-Soto M.A., Aguirre-Avalos G. (2017). Timing, method and discontinuation of hydrocortisone administration for septic shock patients. World J. Crit. Care Med..

[52] Confalonieri M., Urbino R., Potena A., Piattella M., Parigi P., Puccio G., Della Porta R., Giorgio C., Blasi F., Umberger R. (2005). Hydrocortisone Infusion for Severe Community-acquired Pneumonia. Am. J. Respir. Crit. Care Med..

[53] Abdelsalam Rezk N., Mohamed Ibrahim A. (2013). Effects of methyl prednisolone in early ARDS. Egypt. J. Chest Dis. Tuberc..

[54] Weber-Carstens S., Keh D. (2007). Bolus or continuous hydrocortisone—That is the question. Crit. Care.

[55] Loisa P., Parviainen I., Tenhunen J., Hovilehto S., Ruokonen E. (2007). Effect of mode of hydrocortisone administration on glycemic control in patients with septic shock: A prospective randomized trial. Crit. Care.

[56] Winkler M.S., Osuchowski M.F., Payen D., Torres A., Dickel S., Skirecki T. (2022). Renaissance of glucocorticoids in critical care in the era of COVID-19: Ten urging questions. Crit. Care.

[57] National Heart, Lung, and Blood Institute Acute Respiratory Distress Syndrome (ARDS) (2006). Clinical Trials Network. Efficacy and Safety of Corticosteroids for Persistent Acute Respiratory Distress Syndrome. N. Engl. J. Med..

[58] Meduri G.U., Bridges L., Siemieniuk R.A.C., Kocak M. (2018). An Exploratory Reanalysis of the Randomized Trial on Efficacy of Corticosteroids as Rescue Therapy for the Late Phase of Acute Respiratory Distress Syndrome*. Crit. Care Med..

[59] Chen P., Cheng C., Li L., Yu C. (2021). Pneumonia rebound after stopping steroid in a patient with COVID-19: A case report. Respirol. Case Rep..

[60] Nguyen-Ho L. (2022). Disease progression after discontinuation of corticosteroid treatment in a COVID-19 patient with ARDS. Asian Pac. J. Trop. Med..

[61] Murray J.F., Matthay M.A., Luce J.M., Flick M.R. (1988). An Expanded Definition of the Adult Respiratory Distress Syndrome. Am. Rev. Respir. Dis..

[62] Meduri G.U., Headley S., Kohler G., Stentz F., Tolley E., Umberger R., Leeper K. (1995). Persistent Elevation of Inflammatory Cytokines Predicts a Poor Outcome in ARDS. Chest.

[63] Meduri G.U., Tolley E.A., Chrousos G.P., Stentz F. (2002). Prolonged Methylprednisolone Treatment Suppresses Systemic Inflammation in Patients with Unresolving Acute Respiratory Distress Syndrome. Am. J. Respir. Crit. Care Med..

[64] Salton F., Confalonieri P., Torregiani C., Ruaro B., Confalonieri M. (2023). Higher, but Not Too High, Dose Is Only One Determinant of Corticosteroids Treatment Success in Severe COVID-19. Ann. Am. Thorac. Soc..

[65] Meduri G.U., Headley S., Tolley E., Shelby M., Stentz F., Postlethwaite A. (1995). Plasma and BAL Cytokine Response to Corticosteroid Rescue Treatment in Late ARDS. Chest.

[66] Chriguer R.S., Elias L.L.K., da Silva I.M., Vieira J.G.H., Moreira A.C., de Castro M. (2005). Glucocorticoid Sensitivity in Young Healthy Individuals: In Vitro and in Vivo Studies. J. Clin. Endocrinol. Metab..

[67] Cohen J., Pretorius C.J., Ungerer J.P.J., Cardinal J., Blumenthal A., Presneill J., Gatica-Andrades M., Jarrett P., Lassig-Smith M., Stuart J. (2016). Glucocorticoid Sensitivity Is Highly Variable in Critically Ill Patients with Septic Shock and Is Associated with Disease Severity. Crit. Care Med..

[68] Meduri G.U., Golden E., Freire A.X., Taylor E., Zaman M., Carson S.J., Gibson M., Umberger R. (2007). Methylprednisolone Infusion in Early Severe ARDS. Chest.

[69] Van den Akker E.L.T., Koper J.W., Joosten K., de Jong F.H., Hazelzet J.A., Lamberts S.W., Hokken-Koelega A.C. (2009). Glucocorticoid receptor mRNA levels are selectively decreased in neutrophils of children with sepsis. Intensive Care Med..

[70] Müller B., Peri G., Doni A., Landmann R., Pasqualini F., Mantovani A. (2002). High circulating levels of the IL-1 type II decoy receptor in critically ill patients with sepsis: Association of high decoy receptor levels with glucocorticoid administration. J. Leukoc. Biol..

[71] De Kruif M.D., Lemaire L.C., Giebelen I.A., Struck J., Morgenthaler N.G., Papassotiriou J., Elliott P.J., van der Poll T. (2008). The influence of corticosteroids on the release of novel biomarkers in human endotoxemia. Intensive Care Med..

[72] Perren A., Cerutti B., Lepori M., Senn V., Capelli B., Duchini F., Domenighetti G. (2008). Influence of Steroids on Procalcitonin and C-reactive Protein in Patients with COPD and Community-acquired Pneumonia. Infection.

[73] Rinaldi S., Adembri C., Grechi S., De Gaudio A.R. (2006). Low-dose hydrocortisone during severe sepsis: Effects on microalbuminuria. Crit. Care Med..

[74] Wilkinson L., Verhoog N.J.D., Louw A. (2018). Disease- and treatment-associated acquired glucocorticoid resistance. Endocr. Connect..

[75] Rodriguez J.M., Monsalves-Alvarez M., Henriquez S., Llanos M.N., Troncoso R. (2016). Glucocorticoid resistance in chronic diseases. Steroids.

[76] Meduri G.U., Yates C.R. (2004). Systemic Inflammation-Associated Glucocorticoid Resistance and Outcome of ARDS. Ann. N. Y. Acad. Sci..

[77] Koper J.W., van Rossum E.F.C., van den Akker E.L.T. (2014). Glucocorticoid receptor polymorphisms and haplotypes and their expression in health and disease. Steroids.

[78] Bergquist M., Nurkkala M., Rylander C., Kristiansson E., Hedenstierna G., Lindholm C. (2013). Expression of the glucocorticoid receptor is decreased in experimental *Staphylococcus aureus* sepsis. J. Infect..

[79] Indyk J.A., Candido-Vitto C., Wolf I.M., Venkataraman S., Munoz R., Saladino R.A., Witchel S.F., Defranco D.B. (2013). Reduced Glucocorticoid Receptor Protein Expression in Children with Critical Illness. Horm. Res. Paediatr..

[80] Vassiliou A.G., Floros G., Jahaj E., Stamogiannos G., Gennimata S., Vassiliadi D.A., Tsagarakis S., Tzanela M., Ilias I., Orfanos S.E. (2019). Decreased glucocorticoid receptor expression during critical illness. Eur. J. Clin. Investig..

[81] Guerrero J., Gatica H.A., Rodríguez M., Estay R., Goecke I. (2013). Septic serum induces glucocorticoid resistance and modifies the expression of glucocorticoid isoforms receptors: A prospective cohort study and in vitro experimental assay. Crit. Care.

[82] Park J.H., Lee H.K. (2020). Re-analysis of Single Cell Transcriptome Reveals That the NR3C1-CXCL8-Neutrophil Axis Determines the Severity of COVID-19. Front. Immunol..

[83] Van der Voort P.H.J., Gerritsen R.T., Bakker A.J., Boerma E.C., Kuiper M.A., de Heide L. (2003). HDL-cholesterol level and cortisol response to synacthen in critically ill patients. Intensive Care Med..

[84] Yates C.R., Chang C., Kearbey J.D., Yasuda K., Schuetz E.G., Miller D.D., Dalton J.T., Swaan P.W. (2003). Structural Determinants of P-Glycoprotein-Mediated Transport of Glucocorticoids. Pharm. Res..

[85] Okamoto K., Tanaka H., Makino Y., Makino I. (1998). Restoration of the Glucocorticoid Receptor Function by the Phosphodiester Compound of Vitamins C and E, EPC-K1 (l-Ascorbic Acid 2-[3, 4-Dihydro-2, 5, 7, 8-tetramethyl-2-(4, 8, 12-trimethyltridecyl)-2H-1-benzopyran-6-yl Hydrogen Phosphate] Potassium Salt), via a Redox-dependent Mechanism. Biochem. Pharmacol..

[86] Okamoto K., Tanaka H., Ogawa H., Makino Y., Eguchi H., Hayashi S., Yoshikawa N., Poellinger L., Umesono K., Makino I. (1999). Redox-dependent Regulation of Nuclear Import of the Glucocorticoid Receptor. J. Biol. Chem..

[87] Grimes D.S. (2006). Are statins analogues of vitamin D?. Lancet.

[88] Hoepner R., Bagnoud M., Pistor M., Salmen A., Briner M., Synn H., Schrewe L., Guse K., Ahmadi F., Demir S. (2019). Vitamin D increases glucocorticoid efficacy via inhibition of mTORC1 in experimental models of multiple sclerosis. Acta Neuropathol..

[89] Ojaimi S., Skinner N.A., Strauss B.J., Sundararajan V., Woolley I., Visvanathan K. (2013). Vitamin d deficiency impacts on expression of toll-like receptor-2 and cytokine profile: A pilot study. J. Transl. Med..

[90] Padayatty S., Levine M. (2016). Vitamin C: The known and the unknown and Goldilocks. Oral Dis..

[91] Patak P., Willenberg H.S., Bornstein S.R. (2004). Vitamin C Is an Important Cofactor for Both Adrenal Cortex and Adrenal Medulla. Endocr. Res..

[92] Björkhem I., Kallner A., Karlmar K.E. (1978). Effects of ascorbic acid deficiency on adrenal mitochondrial hydroxylations in guinea pigs. J. Lipid. Res..

[93] Pariante C.M., Pearce B.D., Pisell T.L., Sanchez C.I., Po C., Su C., Miller A.H. (1999). The Proinflammatory Cytokine, Interleukin-1α, Reduces Glucocorticoid Receptor Translocation and Function. Endocrinology.

[94] Meduri G.U., Muthiah M.P., Carratù P., Eltorky M., Chrousos G.P. (2005). Nuclear Factor-ĸB- and Glucocorticoid Receptor α- Mediated Mechanisms in the Regulation of Systemic and Pulmonary Inflammation during Sepsis and Acute Respiratory Distress Syndrome. Neuroimmunomodulation.

[95] Bantel H., Schmitz M.L., Raible A., Gregor M., Schulze-Osthoff K. (2002). Critical role of nuclear factor-κB and stress-activated protein kinases in steroid unresponsiveness. FASEB J..

[96] Liu M., Zhu L.J., Zhou Q.G. (2013). Neuronal nitric oxide synthase is an endogenous negative regulator of glucocorticoid receptor in the hippocampus. Neurol. Sci..

[97] Hutchison K.A., Matić G., Meshinchi S., Bresnick E.H., Pratt W.B. (1991). Redox manipulation of DNA binding activity and BuGR epitope reactivity of the glucocorticoid receptor. J. Biol. Chem..

[98] Makino Y., Okamoto K., Yoshikawa N., Aoshima M., Hirota K., Yodoi J., Umesono K., Makino I., Tanaka H. (1996). Thioredoxin: A redox-regulating cellular cofactor for glucocorticoid hormone action. Cross talk between endocrine control of stress response and cellular antioxidant defense system. J. Clin. Investig..

[99] Duma D., Silva-Santos J.E., Assreuy J. (2004). Inhibition of glucocorticoid receptor binding by nitric oxide in endotoxemic rats. Crit. Care Med..

[100] Hakim A., Barnes P.J., Adcock I.M., Usmani O.S. (2013). Importin-7 mediates glucocorticoid receptor nuclear import and is impaired by oxidative stress, leading to glucocorticoid insensitivity. FASEB J..

[101] Farrell R., Kelleher D. (2003). Glucocorticoid resistance in inflammatory bowel disease. J. Endocrinol..

[102] Hearing S.D., Norman M., Smyth C., Foy C., Dayan C.M. (1999). Wide Variation in Lymphocyte Steroid Sensitivity Among Healthy Human Volunteers. J. Clin. Endocrinol. Metab..

[103] Smit P., Russcher H., de Jong F.H., Brinkmann A.O., Lamberts S.W.J., Koper J.W. (2005). Differential Regulation of Synthetic Glucocorticoids on Gene Expression Levels of Glucocorticoid-Induced Leucine Zipper and Interleukin. J. Clin. Endocrinol. Metab..

[104] Oray M., Abu Samra K., Ebrahimiadib N., Meese H., Foster C.S. (2016). Long-term side effects of glucocorticoids. Expert Opin. Drug Saf..

[105] Yuanjing C., Li K., Pu H., Wu T. (2009). Corticosteroids for pneumonia. Cochrane Database Syst. Rev..

[106] Edalatifard M., Akhtari M., Salehi M., Naderi Z., Jamshidi A., Mostafaei S., Najafizadeh S.R., Farhadi E., Jalili N., Esfahani M. (2020). Intravenous methylprednisolone pulse as a treatment for hospitalised severe COVID-19 patients: Results from a randomised controlled clinical trial. Eur. Respir. J..

[107] Tang X., Feng Y.M., Ni J.X., Zhang J.Y., Liu L.M., Hu K., Wu X.Z., Zhang J.X., Chen J.W., Zhang J.C. (2021). Early Use of Corticosteroid May Prolong SARS-CoV-2 Shedding in Non-Intensive Care Unit Patients with COVID-19 Pneumonia: A Multicenter, Single-Blind, Randomized Control Trial. Respiration.

[108] Tomazini B.M., Maia I.S., Cavalcanti A.B., Berwanger O., Rosa R.G., Veiga V.C., Avezum A., Lopes R.D., Bueno F.R., Silva M.V.A.O. (2020). Effect of Dexamethasone on Days Alive and Ventilator-Free in Patients with Moderate or Severe Acute Respiratory Distress Syndrome and COVID-19. JAMA.

[109] Corral-Gudino L., Bahamonde A., Arnaiz-Revillas F., Gómez-Barquero J., Abadía-Otero J., García-Ibarbia C., Mora V., Cerezo-Hernández A., Hernández J.L., López-Muñíz G. (2021). Methylprednisolone in adults hospitalized with COVID-19 pneumonia. Wien. Klin. Wochenschr..

[110] Leistner R., Schroeter L., Adam T., Poddubnyy D., Stegemann M., Siegmund B., Maechler F., Geffers C., Schwab F., Gastmeier P. (2022). Corticosteroids as risk factor for COVID-19-associated pulmonary aspergillosis in intensive care patients. Crit. Care.

[111] Jeronimo C.M.P., Farias M.E.L., Val F.F.A., Sampaio V.S., Alexandre M.A.A., Melo G.C., Safe I.P., Borba M.G.S., Netto R.L.A., Maciel A.B.S. (2021). Methylprednisolone as Adjunctive Therapy for Patients Hospitalized with Coronavirus Disease 2019 (COVID-19; Metcovid): A Randomized, Double-blind, Phase IIb, Placebo-controlled Trial. Clin. Infect. Dis..

[112] Prete A., Bancos I. (2021). Glucocorticoid induced adrenal insufficiency. BMJ.

[113] Nawab Q.U.A., Golden E., Confalonieri M., Umberger R., Meduri U.G. (2011). Corticosteroid treatment in severe community-acquired pneumonia: Duration of treatment affects control of systemic inflammation and clinical improvement. Intensive Care Med..

[114] Mondini L., Salton F., Trotta L., Bozzi C., Pozzan R., Barbieri M., Tavano S., Lerda S., Hughes M., Confalonieri M. (2023). Host-Based Treatments for Severe COVID-19. Curr. Issues Mol. Biol..

[115] WHO (2023). Therapeutics and COVID-19: Living Guideline, 13 January 2023 (WHO/2019-NCoV/Therapeutics/2023.1).

[116] Salton F., Confalonieri P., Campisciano G., Cifaldi R., Rizzardi C., Generali D., Pozzan R., Tavano S., Bozzi C., Lapadula G. (2022). Cytokine Profiles as Potential Prognostic and Therapeutic Markers in SARS-CoV-2-Induced ARDS. J. Clin. Med..

[117] Abani O., Abbas A., Abbas F., Abbas M., Abbasi S., Abbass H., Abbott A., Abdallah N., Abdelaziz A., Abdelfattah M. (2021). Tocilizumab in Patients Admitted to Hospital with COVID-19 (RECOVERY): A Randomised, Controlled, Open-Label, Platform Trial. Lancet.

[118] Coronavirus (COVID-19)|Drugs. https://www.fda.gov/drugs/emergency-preparedness-drugs/coronavirus-covid-19-drugs.

[119] COVID-19 Treatments. https://www.ema.europa.eu/en/human-regulatory/overview/public-health-threats/coronavirus-disease-covid-19/treatments-vaccines/covid-19-treatments.

[120] Kyriazopoulou E., Huet T., Cavalli G., Gori A., Kyprianou M., Pickkers P., Eugen-Olsen J., Clerici M., Veas F., Chatellier G. (2021). Effect of Anakinra on Mortality in Patients with COVID-19: A Systematic Review and Patient-Level Meta-Analysis. Lancet Rheumatol..

[121] Abani O., Abbas A., Abbas F., Abbas J., Abbas K., Abbas M., Abbasi S., Abbass H., Abbott A., Abbott A. (2022). Baricitinib in Patients Admitted to Hospital with COVID-19 (RECOVERY): A Randomised, Controlled, Open-Label, Platform Trial and Updated Meta-Analysis. Lancet.

[122] Grundeis F., Ansems K., Dahms K., Thieme V., Metzendorf M.I., Skoetz N., Benstoem C., Mikolajewska A., Griesel M., Fichtner F. (2023). Remdesivir for the treatment of COVID-19. Cochrane Database Syst. Rev..

[123] Mastruzzo C., Commodari E., Grasso U., La Rosa V.L., Balsamo D., Circo C., Oliveri R. (2023). Early Stage Combination Treatment with Methylprednisolone Pulse and Remdesivir for Severe COVID-19 Pneumonia. Int. J. Environ. Res. Public Health.

[124] Zhu Q., Xu Y., Wang T., Xie F. (2022). Innate and adaptive immune response in SARS-CoV-2 infection—Current perspectives. Front. Immunol..

[125] Gressens S.B., Esnault V., De Castro N., Sellier P., Sene D., Chantelot L., Hervier B., Delaugerre C., Chevret S., Molina J.M. (2022). Remdesivir in combination with dexamethasone for patients hospitalized with COVID-19: A retrospective multicenter study. PLoS ONE.

[126] Tasaka S., Ohshimo S., Takeuchi M., Yasuda H., Ichikado K., Tsushima K., Egi M., Hashimoto S., Shime N., Saito O. (2022). ARDS Clinical Practice Guideline 2021. J. Intensive Care.

[127] Apostolo D., D’Onghia D., Tonello S., Minisini R., Baricich A., Gramaglia C., Patrucco F., Zeppegno P., Acquaviva A., Balbo P.E. (2023). Decreased Gas6 and sAxl Plasma Levels Are Associated with Hair Loss in COVID-19 Survivors. Int. J. Mol. Sci..

[128] Ruan S.Y., Lin H.H., Huang C.T., Kuo P.H., Wu H.D., Yu C.J. (2014). Exploring the heterogeneity of effects of corticosteroids on acute respiratory distress syndrome: A systematic review and meta-analysis. Crit. Care.

[129] Dequin P.F., Meziani F., Quenot J.P., Kamel T., Ricard J.D., Badie J., Reignier J., Heming N., Plantefève G., Souweine B. (2023). Hydrocortisone in Severe Community-Acquired Pneumonia. N. Engl. J. Med..

[130] Blum C.A., Nigro N., Briel M., Schuetz P., Ullmer E., Suter-Widmer I., Winzeler B., Bingisser R., Elsaesser H., Drozdov D. (2015). Adjunct prednisone therapy for patients with community-acquired pneumonia: A multicentre, double-blind, randomised, placebo-controlled trial. Lancet.

[131] Ruaro B., Confalonieri P., Pozzan R., Tavano S., Mondini L., Baratella E., Pagnin A., Lerda S., Geri P., Biolo M. (2022). Severe COVID-19 ARDS Treated by Bronchoalveolar Lavage with Diluted Exogenous Pulmonary Surfactant as Salvage Therapy: In Pursuit of the Holy Grail?. J. Clin. Med..

[132] Russo A., Davoli C., Borrazzo C., Olivadese V., Ceccarelli G., Fusco P., Lazzaro A., Lionello R., Ricchio M., Serapide F. (2022). Clinical Characteristics and Outcome of Hospitalized COVID-19 Patients Treated with Standard Dose of Dexamethasone or High Dose of Methylprednisolone. Biomedicines.

[133] Pelosi P., Tonelli R., Torregiani C., Baratella E., Confalonieri M., Battaglini D., Marchioni A., Confalonieri P., Clini E., Salton F. (2022). Different Methods to Improve the Monitoring of Noninvasive Respiratory Support of Patients with Severe Pneumonia/ARDS Due to COVID-19: An Update. J. Clin. Med..

[134] Baratella E., Bussani R., Zanconati F., Marrocchio C., Fabiola G., Braga L., Maiocchi S., Berlot G., Volpe M.C., Moro E. (2021). Radiological-pathological signatures of patients with COVID-19-related pneumomediastinum: Is there a role for the Sonic hedgehog and Wnt5a pathways?. ERJ Open Res..

